# Supplementary material for: Ethnographic meta-analysis shows that thermoregulation activities predict needle and awl use in North America
Source: PLoS One. 2026 Mar 5;21(3):e0343888. doi: 10.1371/journal.pone.0343888 (PMC12962513; doi:10.1371/journal.pone.0343888)
Supplement: Attachment — Submitted filename: PONE-D-25-39223_reviewer.pdf [file pone.0343888.s002.pdf]

PLOS ONE

Ethnographic meta-analysis shows that thermoregulation activities predict needle and awl use in North America

--Manuscript Draft--

**Manuscript Number:**	PONE-D-25-39223
**Article Type:**	Research Article
**Full Title:**	Ethnographic meta-analysis shows that thermoregulation activities predict needle and awl use in North America
**Short Title:**	North American needle and awl use
**Corresponding Author:**	McKenna Lynn Litynski<br>University of Wyoming<br>Laramie, Wyoming UNITED STATES OF AMERICA
**Keywords:**	needle; awl; temperature relationships; thermoregulation; activity types
**Abstract:**	Needles and awls are common artifacts in the perishable archaeological record. To understand behavioral motivations for their use, this study examines ethnographic activities linked to needles and awls in North America. We hypothesize that thermoregulation would have been the strongest driver of perforator use. Ethnographies from eHRAF World Cultures are examined to evaluate activity types and temperature effects on the prevalence of perforator use. We observe that the sum of non-thermoregulation activities (69%) account for the majority of ethnographic perforator occurrences. Nonetheless, the most prevalent activity identified is clothing production, accounting for 14% of ethnographic observations. We furthermore observe from a series of logistical regression models that for each unit of temperature increase, the probability of perforator observation decreases by a multiplicative factor of 0.13±0.00, with a 61±14% probability of observing perforators at the coldest temperatures and 43±10% probability at the warmest. These results support the hypothesis that thermoregulation, particularly clothing manufacture, was a major driver of perforator tool use while simultaneously revealing that such tools were deployed in a wide array of activities. Such findings provide insight into the environmental and socio-cultural factors that influenced the use of perforators and thus inform our understanding of an artifact class that is increasingly observed in the archaeological record.
**Order of Authors:**	McKenna Lynn Litynski
	Sean Field
	Randall Haas
**Additional Information:**	
**Question**	**Response**
**Financial Disclosure**<br><br>Enter a financial disclosure statement that describes the sources of funding for the work included in this submission. Review the submission guidelines for detailed requirements. View published research articles from *PLOS ONE* for specific examples.<br><br>This statement is required for submission and **will appear in the published article** if the submission is accepted. Please make	The author(s) received no specific funding for this work.

Powered by Editorial Manager® and ProduXion Manager® from Aries Systems Corporation

sure it is accurate.<br><br>**Funded studies**<br>Enter a statement with the following details:<br>• Initials of the authors who received each award<br>• Grant numbers awarded to each author<br>• The full name of each funder<br>• URL of each funder website<br>• Did the sponsors or funders play any role in the study design, data collection and analysis, decision to publish, or preparation of the manuscript?<br><br>Did you receive funding for this work?	
**Competing Interests**<br><br>Use the instructions below to enter a competing interest statement for this submission. On behalf of all authors, disclose any competing interests that could be perceived to bias this work—acknowledging all financial support and any other relevant financial or non-financial competing interests.<br><br>This statement is required for submission and **will appear in the published article** if the submission is accepted. Please make sure it is accurate and that any funding sources listed in your Funding Information later in the submission form are also declared in your Financial Disclosure statement.<br><br>View published research articles from *PLOS ONE* for specific examples.	The authors have declared that no competing interests exist.

Powered by Editorial Manager® and ProduXion Manager® from Aries Systems Corporation

**NO authors have competing interests**<br><br>Enter: *The authors have declared that no competing interests exist.*<br><br>**Authors with competing interests**<br><br>Enter competing interest details beginning with this statement:<br><br>*I have read the journal's policy and the authors of this manuscript have the following competing interests: [insert competing interests here]*<br><br>* typeset	
**Ethics Statement**<br><br>Enter an ethics statement for this submission. This statement is required if the study involved:<br><br>• Human participants<br>• Human specimens or tissue<br>• Vertebrate animals or cephalopods<br>• Vertebrate embryos or tissues<br>• Field research<br><br>Write "N/A" if the submission does not require an ethics statement.<br><br>General guidance is provided below. Consult the submission guidelines for detailed instructions. **Make sure that all information entered here is included in the Methods section of the manuscript.**	N/A

Powered by Editorial Manager® and ProduXion Manager® from Aries Systems Corporation

**Format for specific study types**<br><br>**Human Subject Research (involving human participants and/or tissue)**<br>• Give the name of the institutional review board or ethics committee that approved the study<br>• Include the approval number and/or a statement indicating approval of this research<br>• Indicate the form of consent obtained (written/oral) or the reason that consent was not obtained (e.g. the data were analyzed anonymously)<br><br>**Animal Research (involving vertebrate animals, embryos or tissues)**<br>• Provide the name of the Institutional Animal Care and Use Committee (IACUC) or other relevant ethics board that reviewed the study protocol, and indicate whether they approved this research or granted a formal waiver of ethical approval<br>• Include an approval number if one was obtained<br>• If the study involved *non-human primates*, add *additional details* about animal welfare and steps taken to ameliorate suffering<br>• If anesthesia, euthanasia, or any kind of animal sacrifice is part of the study, include briefly which substances and/or methods were applied<br><br>**Field Research**<br><br>Include the following details if this study involves the collection of plant, animal, or other materials from a natural setting:<br>• Field permit number<br>• Name of the institution or relevant body that granted permission	
**Data Availability**<br><br>Authors are required to make all data underlying the findings described fully available, without restriction, and from the time of publication. PLOS allows rare exceptions to address legal and ethical concerns. See the PLOS Data Policy and FAQ for detailed information.	Yes - all data are fully available without restriction

A Data Availability Statement describing where the data can be found is required at submission. Your answers to this question constitute the Data Availability Statement and **will be published in the article**, if accepted.<br><br>**Important:** Stating 'data available on request from the author' is not sufficient. If your data are only available upon request, select 'No' for the first question and explain your exceptional situation in the text box.<br><br>Do the authors confirm that all data underlying the findings described in their manuscript are fully available without restriction?	
**Describe where the data may be found in full sentences. If you are copying our sample text, replace any instances of XXX with the appropriate details.**<br><br>• If the data are **held or will be held in a public repository**, include URLs, accession numbers or DOIs. If this information will only be available after acceptance, indicate this by ticking the box below. For example: *All XXX files are available from the XXX database (accession number(s) XXX, XXX.).*<br>• If the data are all contained **within the manuscript and/or Supporting Information files**, enter the following: *All relevant data are within the manuscript and its Supporting Information files.*<br>• If neither of these applies but you are able to provide **details of access elsewhere**, with or without limitations, please do so. For example:<br><br>*Data cannot be shared publicly because of [XXX]. Data are available from the XXX Institutional Data Access / Ethics Committee (contact via XXX) for researchers who meet the criteria for access to confidential data.*<br><br>*The data underlying the results presented in the study are available from (include the name of the third party*	R code associated with this manuscript can be found at this github link: https://github.com/mlitynsk/NA_bonetools<br><br>Currently, this github repository is private but will be made public upon acceptance of the manuscript for publication.<br><br>All other relevant data are within the manuscript and its Supporting Information files.

Powered by Editorial Manager® and ProduXion Manager® from Aries Systems Corporation

and contact information or URL).<br>• This text is appropriate if the data are owned by a third party and authors do not have permission to share the data.<br><br>* typeset	
Additional data availability information:	

Main Manuscript
Click here to access/download;Manuscript;Main Manuscript_PLoS One.docx

Full title: **Ethnographic meta-analysis shows that thermoregulation activities predict needle and awl use in North America**

Short title: **North American needle and awl use**

McKenna L. Litynski, Sean Field, and Randall Haas

DO NOT CITE IN ANY CONTEXT WITHOUT PERMISSION OF THE AUTHORS

McKenna L. Litynski: Department of Anthropology, University of Wyoming, Laramie, WY, USA 82071 (mlitynsk@uwyo.edu, corresponding author)

Sean Field: School of Computing and Department of Anthropology, University of Wyoming, Laramie, WY, USA 82071

Randall Haas: Department of Anthropology, University of Wyoming, Laramie, WY, USA 82071

Abstract

Needles and awls are common artifacts in the perishable archaeological record. To understand behavioral motivations for their use, this study examines ethnographic activities linked to needles and awls in North America. We hypothesize that thermoregulation would have been the strongest driver of perforator use. Ethnographies from eHRAF World Cultures are examined to evaluate activity types and temperature effects on the prevalence of perforator use. We observe that the sum of non-thermoregulation activities (69%) account for the majority of ethnographic perforator occurrences. Nonetheless, the most prevalent activity identified is clothing production, accounting for 14% of ethnographic observations. We furthermore observe from a series of logistical regression models that for each unit of temperature increase, the probability of perforator observation decreases by a multiplicative factor of 0.13±0.00, with a 61±14% probability of observing perforators at the coldest temperatures and 43±10% probability at the warmest. These results support the hypothesis that thermoregulation, particularly clothing manufacture, was a major driver of perforator tool use while simultaneously revealing that such tools were deployed in a wide array of activities. Such findings provide insight into the environmental and socio-cultural factors that influenced the use of perforators and thus inform our understanding of an artifact class that is increasingly observed in the archaeological record.

Keywords: needle, awl, temperature relationships, thermoregulation, activity types

Introduction

Perforator tools made from bone, including needles and awls, are observed in Paleolithic perishable assemblages (Pelton et al. 2024; Wang et al. 2025). Such tools offer critical insight into how early human populations solved the adaptive challenges of Late Pleistocene and Early Holocene global expansion. Needles consist of a long groove and a point/tip and are meant to be passed through a material, while awls increase in width away from the tip but do not have an eye or groove and are not passed completely through the medium (Brietbrode 1983; Pelton 2019). Both tool types have been used by humans for thousands of years, with bone awls dating to as early as ~75 ka (d'Errico 2018; d'Errico et al. 2012; Henshilwood et al. 2001) and needles dating to at least 45 ka (d'Errico 2018). At first glance, perforators might seem to be tools for producing and maintaining leather clothing and housing, which allowed Paleolithic populations to enter and survive cold Pleistocene environments (d'Errico et al. 2018; Gilligan 2010; Gilligan et al. 2024; Lyman 2015; Osborn 2014). However, ethnographic observations suggest that such tools also served a variety of other purposes unrelated to thermoregulation. For example, perforators are commonly used in the production of basketry, tattooing, and wood working (De Laguna 1972; Densmore 1929; Breitborde 1983; Bates 2022; Gijn 2006; Jenness 1949; Keddie 2016; McIlwraith 1948). The extent to which perforator tools can be confidently linked to thermoregulatory activities or other purposes remains unclear, limiting our understanding of how influential these tools were in shaping past human migration into cold environments.

This paper approaches the problem using ethnographic accounts of North American needle and awl use from the eHRAF World Cultures database. We are interested in determining the extent to which needles and awls were created and used for manufacturing thermoregulation items or alternative purposes.

Under a thermoregulation framework, cold climates compel humans to invest in thermoregulatory technologies that often include perforator tools. This model makes two basic empirically testable predictions for the ethnographic record. First, we should expect that perforator tools are most often associated with thermoregulation activities in the ethnographic record. Second, it anticipates that environmental temperatures predict the use of perforator tools. In other words, the frequency of perforator tool observations in the ethnographic record ought to be greater in cold climates than in warm climates. Such predictions align with Osborn's (2014) observation that a 1,300-year cold snap between 12.9-11.6 cal. ka known as the Younger Dryas Cold Event significantly increased the importance of thermoregulatory technology such as the manufacture of tailored skin clothing and thus inflated the production of needles for sewing.

Although there is good reason to suspect that thermoregulation was the major driver of perforator technology, qualitative observation of ethnographic literature shows that needles and awls commonly serve a variety of functional and symbolic purposes outside of the realm of thermoregulation. Examples of such needle and awl functions include tattooing human skin, beadwork, fishing, basketry, woodwork, and serving as gifts for ceremonies (Bates 2022; Breitborde 1983; De Laguna 1972; Densmore 1929; Gijn 2006; Jenness 1949; Keddie 2016; McIlwraith 1948). When used in these capacities, needles and awls are not limited to cold environments.

Moreover, while previous research shows that perforators figure prominently in the production of cold-weather clothing, at least one prominent counter example shows that such technologies are not requisite for cold-climate adaptation. Namely, Garvey (2021:105) highlights how ethnographic Patagonian populations that inhabited cold polar environments did not make tailored leather clothing nor is there evidence of needles, awls, or microblades in the Late

Pleistocene archaeological deposits of Patagonia. The lack of evidence for bone perforator tools suggests individuals may have had alternative physiological and behavioral adaptations to the cold, and the colonization of this region during the Late Pleistocene was likely slow and thus the technology and knowledge to manufacture fitted clothing was lost (Garvey 2021:108).

These varied examples highlight that while perforator tools are linked to thermoregulation activities, the extent to which this is so remains unclear. Such tools likely serve as multi-tools for any combination of activities. The two models of perforator use represent lenses through which we can understand the extent to which needles and awls were used in cold stress adaptations or other applications.

The extent to which climatic conditions in North America drove thermoregulatory technologies and thus the use of perforation tools remains unclear. At one extreme, we could suppose that environmental temperatures exerted a strong effect on the use of such tools. At the other extreme, we could suppose that some other combination of perforator functions overwhelm any climatic effects. The answer has implications for our interpretation of bone perforators in the archaeological record and thus our understanding of human behavior more generally, especially global migrations. The purpose of this analysis is to determine the magnitude of climatic and other effects on the use of perforator tools among ethnographic societies.

Materials and Methods

This study evaluates two predictions derived from the working model. First, the model anticipates that the majority of ethnographic observations of perforator tools (including needles and awls) will primarily be associated with thermoregulatory activities including clothing, shelter, bedding, and blanket manufacture and maintenance. Second, the model anticipates that

the probability of observing the use of perforator tools will increase in colder environments. Ethnographic observations of perforator use in North America are used to evaluate these predictions. We select North America as a case study for understanding perforator tool use based on the ability to obtain data from Indigenous groups spanning extremes of cold and warm conditions at a continental scale. Additionally, the ethnographic observations, unlike archaeological observations, afford a view of the perishable toolkits and behavioral adaptations associated with perforator tools. Here we focus on an ethnographic approach, remaining alert to its analytical limitations and with an eye toward a complimentary archaeological approach in the future. This section describes creating the perforator tool database, operationalization of environmental temperature data, and analytical methods of evaluating tool use types and environmental temperature relationships.

Creating the Perforator Tool Database

The ethnographies compiled to test these predictions are from the eHRAF World Cultures (electronic Human Relations Area Files) database. The eHRAF database provides access to ethnographic texts (Fischer and Ember 2018) from over 300 Indigenous and ethnic groups located in a variety of regions associated with all possible historical climate zones across North America.

We first search all eHRAF records for the terms, “needle” and “awl” (Appendix A). For each record mentioning needles or awls, the tool type, publication citation, page number, activity type (e.g. clothing, blankets, tattooing, basketry, mats, etc.), activity classification (e.g. thermoregulatory or alternative), and perforator material are recorded. Activity types related to the creation or maintenance of materials likely to be used for thermoregulatory purposes (e.g. blankets, clothing, shelter, bedding, mats, etc.) are classified as “thermoregulatory” and all

activities related to materials not directly related to thermoregulation (e.g. basketry, ceremonies, tattooing, storytelling, fishing, etc.) are classified as “alternative”. If a given tool is associated with multiple activities by the same eHRAF source, then each activity is recorded as a separate observation.

We remain alert to several limitations and biases that come with deriving data from eHRAF World Cultures including the impact of ethnocentrism; excluding certain code words such as “punch” that may have yielded additional occurrences of perforator tools; limitations in the number of ethnographic texts included in eHRAF; and inherent gender bias that can skew the perspectives, experiences, and information shared in the ethnographic record (Divale 1976; Slocum 1975; Watts et al. 2021). Though it is important to recognize these limitations and biases in interpreting ethnographic data, the coded data and the temperature trends associated with perforator tool use remains relevant and useful to understanding the wide variety of uses for needles and awls. Furthermore, to limit the biases that accompany use of eHRAF World Cultures such as the presentation of “too much material” (Fischer and Ember 2018:328) and limitations in Indigenous groups associated with eHRAF, we qualitatively evaluate geographic data structure by plotting our database in space and select search terms specific to perforator tools.

Environmental Temperature Data

Our analysis requires the assignment of a temperature variable to each ethnographic case. We use the average minimum temperature of the coldest month (MTCM) to capture thermoregulatory pressures that a given ethnographic group likely experienced. This proxy seems particularly salient relative to common alternative proxies such as effective temperature (Binford 1980; Kelly 2013; Loffler 2016) because MTCM quantifies the extreme or limiting

environmental conditions that impact people's ability to survive in cold environments and thus the dependence of clothing for human survival. We obtain MTCM data from WorldClim, a database consisting of high-resolution historical weather and climate information on a global scale presented in geographic format (i.e. GeoTiff). The MTCM for each ethnographic group in the database is based on the geographic centroid of the group's territory based on eHRAF World Cultures, with MTCM data derived from bioclimatic variable BIO6 from WorldClim (Figure 1). This included groups associated with perforator tool technologies and those with no mention of needles/awls ethnographically. Non-Indigenous cultural groups within eHRAF were not included in this study. All geostatistical operations are performed by ArcGIS.

Figure 1: Heat map featuring the average minimum temperature of the coldest month across North America and geographical centroids of 59 Indigenous groups territories available in eHRAF World Cultures.

Statistical Analysis

To test the prediction that the majority of perforator tools were used for thermoregulation, the total number of ethnographic documents within the "thermoregulation" and "alternative" categories were converted to percentages with the exception that the proportion of thermoregulatory activities should exceed that of other activities. A chi-square test is used to evaluate statistical significance of observed differences in proportions.

To assess the effect of MTCM on perforator tool occurrences, we conduct a series of logistic regression models. The first model examines the effect of MTCM on the frequency of perforator occurrences per ethnographic record. The second model examines the effect of MTCM on the frequency of thermoregulation perforator activities per ethnographic record. The third model evaluates the effect of MTCM on the frequency of non-thermoregulatory perforator activities per ethnographic record. Additional models evaluate the effect of MTCM on the

frequency of major activity types. For all models, we account for differential representation of documents by incorporating the total frequency of documents associated with each Indigenous group as a random-effect variable. Error terms are reported as 95% confidence intervals.

Results

The eHRAF database includes ethnographic observations from 59 North American Indigenous cultural groups. Of these, needle use is observed in the ethnographies of 55 groups (93%) represented in 278 documents (Supplemental Appendix A). Awl use is observed among 54 groups (92%) within 273 literature sources. Of the 467 individual documents examined, we record 1,191 separate needle or awl observations in the ethnographic record. This includes instances where multiple activities were associated with the same eHRAF document. Here we use these observations to assess the extent to which thermoregulatory and other activities anticipate the use of perforator tools in the ethnographic record of North America.

Tool Function

Two-hundred and twenty-three mentions of needles and/or awls in the eHRAF ethnographic record fall under the generic "Toolkit" category and thus cannot be assigned to a specific activity. We record a total of 92 distinct activities. The most frequent activity type observed in association with perforator tools is "Clothing" with 133 observations (Table 1). The second most frequent activity is ceremonies or rituals with 76 observations. Other activities with more than 20 observations include basketry (61), tattooing (59), shoes (48), piercings (37), storytelling (35), trading (35), mats (33), shelter (31), medical (29), snowshoes (26), blankets (21), and fishing (20; Table 1; Figure 2). When considering the frequency of thermoregulation activities (n=301, 31%) and those activities associated with alternative purposes (n=665, 69%),

we observe a statistically significant difference (X^2=137, *d*f=1, *p*=<0.01). We therefore observe that clothing manufacture and maintenance is the most prominent single activity associated with perforator tools. However, the occurrence rate of alternative activities, in aggregate, is approximately double that of thermoregulatory activities. These observations indicate that while clothing production and thus thermoregulation do largely motivate the use of perforator tools, alternative activities are also important.

Figure 2: Bar graph showcasing activities associated with needles and awls within the eHRAF World Cultures database along with the number of instances each activity was recorded as part of the meta-analysis. Only those activities observed on 20 or more occasions in the ethnographic record are featured in this graph.

Table 1: Table featuring the various perforator tool uses, the number of occurrences within the ethnographic record unique to awls and needles individually, the total count of both needles and awls within the ethnographic record, and whether these uses are related to thermoregulation or if these tools were used for alternative purposes. The table also provides a description of how each use type has been classified within the ethnographic record.

Usage category	Thermo-regulation?	Awl (count)	Awl (%)	Needle (count)	Needle (%)	Total Tool (count)	Total Too[illegible] (%)
Toolkit	NA*	136	NA*	87	NA*	223	NA
Clothing	Yes	62	12.60%	71	14.98%	133	13.77%
Ceremony or Ritual	No	41	8.33%	35	7.38%	76	7.87%
Baskets	No	52	10.57%	9	1.90%	61	6.31%
Tattooing	No	11	2.24%	48	10.13%	59	6.11%
Shoes	Yes	29	5.89%	19	4.01%	48	4.97%
Piercing	No	21	4.27%	16	3.38%	37	3.83%
Storytelling	No	25	5.08%	10	2.11%	35	3.62%
Trading	No	25	5.08%	10	2.11%	35	3.62%
Mats	Yes	5	1.02%	28	5.91%	33	3.42%
Shelter	Yes	16	3.25%	15	3.16%	31	3.21%
Medical	No	1	0.20%	28	5.91%	29	3.00%
Snowshoes	Yes	6	1.22%	20	4.22%	26	2.69%
Blankets	Yes	6	1.22%	15	3.16%	21	2.17%
Fishing	No	3	0.61%	17	3.59%	20	2.07%
Foodways	No	11	2.24%	5	1.05%	16	1.66%
Beadwork	No	2	0.41%	13	2.74%	15	1.55%
Bags	No	6	1.22%	7	1.48%	13	1.35%

Boat Covering	No	6	1.22%	7	1.48%	13	1.35%
Name	No	13	2.64%	0	0.00%	13	1.35%
Gifts	No	6	1.22%	6	1.27%	12	1.24%
Hunting	No	2	0.41%	10	2.11%	12	1.24%
Magic	No	2	0.41%	10	2.11%	12	1.24%
Punishment	No	2	0.41%	10	2.11%	12	1.24%
Scratching	No	0	0.00%	12	2.53%	12	1.24%
Gaming	No	9	1.83%	2	0.42%	11	1.14%
Metalwork	No	11	2.24%	0	0.00%	11	1.14%
Dance	No	7	1.42%	3	0.63%	10	1.04%
Quillwork	No	9	1.83%	1	0.21%	10	1.04%
Art	No	9	1.83%	0	0.00%	9	0.93%
Belief	No	3	0.61%	5	1.05%	8	0.83%
Jewelry	No	6	1.22%	2	0.42%	8	0.83%
Bedding	Yes	1	0.20%	7	1.48%	8	0.83%
Music	No	6	1.22%	1	0.21%	7	0.72%
Birch Bark Vessels	No	5	1.02%	1	0.21%	6	0.62%
Birch Bark Canoe	No	5	1.02%	0	0.00%	5	0.52%
Wampum belt	No	3	0.61%	2	0.42%	5	0.52%
Weapon	No	4	0.81%	1	0.21%	5	0.52%
Burial	No	1	0.20%	3	0.63%	4	0.41%
Drilling	No	4	0.81%	0	0.00%	4	0.41%
Quivers	No	3	0.61%	1	0.21%	4	0.41%
Saddles	No	4	0.81%	0	0.00%	4	0.41%
Silver beads	No	4	0.81%	0	0.00%	4	0.41%
Suicide	No	4	0.81%	0	0.00%	4	0.41%
Compass	No	1	0.20%	2	0.42%	3	0.31%
Head Ornament	No	3	0.61%	0	0.00%	3	0.31%
Pressure Flaker /Flintknapping	No	3	0.61%	0	0.00%	3	0.31%
Ropes	No	2	0.41%	1	0.21%	3	0.31%
Burden Strap	No	0	0.00%	2	0.42%	2	0.21%
Children Fighting	No	2	0.41%	0	0.00%	2	0.21%
Child's play	No	1	0.20%	1	0.21%	2	0.21%
Cups	No	1	0.20%	1	0.21%	2	0.21%
Gun Caps	No	1	0.20%	1	0.21%	2	0.21%
Horse Equipment	No	2	0.41%	0	0.00%	2	0.21%
Mask	No	0	0.00%	2	0.42%	2	0.21%
Medicine Bundle	No	1	0.20%	1	0.21%	2	0.21%
Politics	No	2	0.41%	0	0.00%	2	0.21%
Quirts	No	2	0.41%	0	0.00%	2	0.21%
Torture	No	2	0.41%	0	0.00%	2	0.21%
Utensils	No	1	0.20%	1	0.21%	2	0.21%

Accident	No	1	0.20%	0	0.00%	1	0.10%
Astrology	No	0	0.00%	1	0.21%	1	0.10%
Basins	No	0	0.00%	1	0.21%	1	0.10%
Buckets	No	0	0.00%	1	0.21%	1	0.10%
Bone Working	No	1	0.20%	0	0.00%	1	0.10%
Bow Backings	No	1	0.20%	0	0.00%	1	0.10%
Bowstring	No	1	0.20%	0	0.00%	1	0.10%
Button	No	1	0.20%	0	0.00%	1	0.10%
Carrying Net	No	0	0.00%	1	0.21%	1	0.10%
Chests	No	0	0.00%	1	0.21%	1	0.10%
Comb	No	0	0.00%	1	0.21%	1	0.10%
Concha	No	1	0.20%	0	0.00%	1	0.10%
Curing Sick Sheep	No	0	0.00%	1	0.21%	1	0.10%
Decorated Birch Bark	No	0	0.00%	1	0.21%	1	0.10%
Dog Care	No	0	0.00%	1	0.21%	1	0.10%
Handkerchiefs	No	0	0.00%	1	0.21%	1	0.10%
Invective	No	1	0.20%	0	0.00%	1	0.10%
Menstruation Sequestration	No	1	0.20%	0	0.00%	1	0.10%
Plates	No	0	0.00%	1	0.21%	1	0.10%
Prayer Stick	No	1	0.20%	0	0.00%	1	0.10%
Sacrifice	No	1	0.20%	0	0.00%	1	0.10%
Seat	No	1	0.20%	0	0.00%	1	0.10%
Sieve	No	1	0.20%	0	0.00%	1	0.10%
Sled	Yes	1	0.20%	0	0.00%	1	0.10%
Stone pipe	No	1	0.20%	0	0.00%	1	0.10%
Stripping Bark	No	1	0.20%	0	0.00%	1	0.10%
Supernatural	No	0	0.00%	1	0.21%	1	0.10%
Vision	No	1	0.20%	0	0.00%	1	0.10%
Wood Working	No	1	0.20%	0	0.00%	1	0.10%

*NA is assigned to the "Toolkit" category based on the generalized nature of such accounts in the ethnographic record. It is not possible to assign "Toolkit" counts to either thermoregulation or alternative use, and we did not include this use category in GLM models or percentage contributions considered in this paper.

We furthermore observe clear differences in the activity types performed with needles and awls, often but not always in obvious ways. For example, there are more observations of awls used for ceremonies/rituals, baskets, shoes, piercings, storytelling, and instances of trading compared to needles (see Table 1). Needles are used more frequently for clothing manufacture,

tattooing, mats, medical use, snowshoes, blankets, and fishing (see Table 1). Both perforator tool types are represented in relatively equivalent proportions for manufacturing shelter from both plant and animal materials.

Perforators and Temperature

The MTCM temperatures for 59 cultural groups under consideration range between -35.5°C and +12.9°C. The logistical regression results indicate that the average probability of observing ethnographic perforator tools decreases by a multiplicative factor of 0.02±0.004 for every degree Celsius of temperature increase ($p < 0.01$; Figure 3; Table 2). For example, whereas at an MTCM -35.5 °C there is a 61±14% probability of observing perforator tools ethnographically, there is only a 43±10% at an MTCM of +12.9°C. When considered separately, the average probability of observing needles decreases by multiplicative factor of 0.02±0.01 for every unit of temperature increase ($p <0.01$; see Figure 3; see Table 2). In other words, there is a 41±14% chance of observing a bone needle among ethnographic accounts of groups living in regions where MTCM reaches -35.5 °C compared with a 27±10% probability of observing needles among groups living where MTCM reaches +12.9°C. Similarly, the average probability of observing awls decreases by 0.02±0.01 for every unit of temperature increase ($p < 0.01$; see Figure 3; see Table 2). This means that in regions where MTCM reaches -35.5°C, there is a 47±16% chance of observing an awl relative to a 16±11% chance of observing an awl among ethnographic group regions where MTCM is at +12.9°C. These results show that lower temperatures predict increased likelihood of perforator use regardless of tool type but that temperature effects are stronger on awl use compared with needle use.

Figure 3: Paneled figure featuring several best fit logistic regression models demonstrating the relationship between the WorldClim minimum temperature of the coldest month (°C) and the probability of observing a perforator tool within the eHRAF ethnographic database. Observations

of perforator tools within ethnographic text are noted by the black open circles, in which those circles appearing at 1 indicate present while circles appearing at 0 indicate the absence of perforator tools within a given ethnographic document. The red line represents the best fit logistic regression model and the dotted lines showcase 95% confidence intervals for each model. Panel A features the results of a logistic regression model considering all perforator tools. Panel B considered only those perforator tools used for alternative purposes. Panel C took into account only those perforators used for thermoregulation purposes. Finally, we evaluated the relationship between MTCM and the probability of observing awls (Panel D) and needles (Panel E) in the ethnographic record.

Table 2: Table featuring the results of logistic regression models undertaken for this study. These models considered the relationship between temperature and perforator tool occurrences in the ethnographic record. The minimum temperature of the coldest month is the constant variable across all featured models.

**Model**	**Estimate**	**Standard Error**	**Z-Value**	**P-Value**	**Tool Occurrence Sample Size**
Perforator Tool Count	-0.016	0.004	-3.500	< 0.01***	n=1,191
Needle Count	-0.014	0.005	-2.632	< 0.01***	n=563
Awl Count	-0.020	0.006	-3.308	< 0.01***	n=628
Perforator Tool Count (Thermoregulation Use)	-0.029	0.007	-4.057	< 0.01***	n=301
Perforator Tool Count (Alternative Use)	-0.007	0.005	-1.246	0.213	n=665
Perforator Tool Count (Tattooing Usage)	-0.016	0.015	-1.076	0.282	n=59
Perforator Tool Count (Basketry Usage)	0.085	0.017	4.858	< 0.01***	n=61
Perforator Tool Count (Clothing Usage)	-0.031	0.010	-3.140	< 0.01***	n=133
Perforator Tool Count (Ceremony/Ritual Usage)	0.007	0.014	0.479	0.632	n=76
Perforator Tool Count (Piercing Usage)	-0.026	0.018	-1.407	0.159	n=37
Perforator Tool Count (Shoes Usage)	-0.046	0.016	-2.956	0.003*	n=48
=Perforator Tool Count (Storytelling Usage)	0.004	0.020	0.174	0.862	n=35
Perforator Tool Count (Mats Usage)	0.051	0.022	2.306	0.021*	n=353
Perforator Tool Count (Shelter Usage)	-0.017	0.020	-0.832	0.405	n=31

Perforator Tool Count (Medical Usage)	-0.010	0.021	-0.462	0.644	n=29
Perforator Tool Count (Snowshoes Usage)	-0.111	0.019	-5.801	< 0.01***	n=26
Perforator Tool Count (Blankets Usage)	-0.009	0.026	0.333	0.739	n=21
Perforator Tool Count (Fishing Usage)	-0.091	0.021	-4.387	< 0.01***	n=20

*p-value is less than 0.05
**p-value is less than 0.001
***p-value is less than 0.0001

Logistic regression model is also used to assess the effects MTCM on perforator tools classified as “thermoregulation” versus those classified as “alternative purposes.”. We find that the probability of observing perforator tools used for thermoregulatory purposes decreases by 0.03±0.01 for every unit of temperature increase ($p < 0.01$). The probability of observing a perforator tool drops from 35±17% where MTCM is -35.5°C to 12.7±7% among accounts where MTCM reaches +12.9°C. In contrast, we do not detect a statistically significant relationship between temperature and the prevalence of perforator tools used for alternative purposes. (p=0.21 ; Figure 3; Table 2).

Analysis of tools used for several other distinct activity types shows strong negative relationships between temperature and occurrences of needles or awls. These activities include the manufacture or use of shoes and snowshoes, as well as fishing related activities ($p < 0.01$, Table 2). Other “alternative” uses of needles and awls do not appear to be related to temperatures. Examples of such activities with no significant relationship between temperature and perforator tools occurrences include tattooing (p=0.28), ceremonies or rituals (p=0.63), piercing (p=0.16), storytelling (p=0.86), medical use (p=0.64), shelter (p=0.41), and blankets (p=0.74; Table 2). We find a significant positive effect of MTCM on basketry ($p < 0.01$) and

mats (p=0.02), indicating that the presence of perforator tools increase in warmer temperatures (Table 2). These are unanticipated relationships, which we discuss below.

Discussion

Ultimately motivated to advance our understanding of archaeological perforator tools, this analysis began by asking whether environmental temperature influences the prevalence of perforator tool uses in the ethnographic record, and to what extent needles and awls are associated with thermoregulation activities compared with alternative uses. The working model anticipated that thermoregulation activities would be the primary driver of perforator tool use. We hypothesized that needles and awls would be observed more frequently among ethnographic cultures living in colder environments as opposed to warmer climates. These predictions were tested by examining perforator tool prevalence and use types within the eHRAF ethnographic record, and comparing those observations with environmental temperature data (WorldClim MTCM) associated with 59 Indigenous groups represented in the eHRAF World Cultures database. The representation of perforator tools among 100% of the groups considered in this study highlights the importance of this technology on a continental scale.

The results of the meta-analysis fail to support the initial prediction that the majority of occurrences of needles and awls in the eHRAF World Cultures database would be associated with thermoregulation. Rather, non-thermoregulatory activities significantly outnumber thermoregulatory activities indicating the importance of needles and awls for alternative functions, most notably ceremonies/rituals, basketry, tattooing, piercing, discussions of needles or awls in storytelling, trading, and fishing (see Supplemental Appendix A for more activity types). Nonetheless, the manufacture of clothing is the single most prevalent activity associated with perforator tools with 133 ethnographic observations. Moreover, six out of the eight

thermoregulation activities associated with perforator tools occur in the top 20 coded categories, with four of those within the top 10 most frequently cited categories, underscoring the central importance of thermoregulation in motivating the use of perforator tools. We therefore find general support for the predicted association between thermoregulatory activities and perforator use while also observing a wide range of non-thermoregulatory activities.

The data furthermore support the second prediction derived from the working model that the probability of observing perforator tools in the eHRAF World Cultures ethnographic record would be negatively related to temperature. This hypothesis holds true when needles and awls are compared with MTCM data in separate regression models, and when considering those tools used specifically for thermoregulation. As expected, this trend is not observed when considering the frequencies of perforator tools used for alternative purposes.

The finding of a clear relationship between temperature and perforator use in the ethnographic record reveals the importance of such tools among societies living in cold environments. Yet, a different perspective is required to explain the high frequency of alternative activities observed such as tattooing, medical applications, basketry, etc. One potential link may follow from the ideas of cumulative culture, defined as “the modification, over multiple transmission episodes, of cultural traits (behavioral patterns transmitted through social learning) resulting in an increase in the complexity or efficiency of those traits” (Dean et al. 2014:288). In the context of perforator tools, it is possible that the primary motivator behind needles and awls included the manufacture of clothing and other thermoregulation material culture items. Over generations of cultural transmission episodes, the importance, efficiency, and multipurpose nature of these objects became recognized. Future research has the potential to further validate if cumulative culture theory was a primary contributor to the results presented in this study. Below,

we consider several of the different activity types observed before concluding with additional implications for the archaeological record.

Use Types and Temperature

As expected, perforator tools used to make several thermoregulation items like clothing, shoes, and snowshoes are used more commonly among colder environments. Surprisingly, there is no relationship between temperature and other thermoregulation items such as shelter or blankets. While sample size could be driving these outcomes, it is important to consider that shelter can offer shade to alleviate the effects of hot environments in addition to providing a climate-controlled space in colder landscapes (Pelton 2019). Additionally, blankets can be other deeply rooted in sociocultural phenomena such as ceremonies, rituals, dances and rituals, horse blankets, social status, infant care, trade, and general comfort (Blomberg 2016; Khau 2012; Lycett 2020; Pearlstein 2008; Whitaker 2004; see Supplemental Appendix A). Blankets can also serve as a form of covering to provide shade and protect the skin from sun exposure, therefore alleviating the effects of warm stress.

Mats serve a variety of purposes including bedding, floormats, cushions for sitting, home décor, or used on the walls of housing structures. We anticipated that mats would be more prevalent in cold environments where they serve as a tool to reduce heat loss via thermal conditions with the ground. Consider, for example, backpackers who almost invariably transport and sleep on ground pads. However, housing structures are applicable to maintaining thermoneutrality in both warm and cold environments, and nighttime temperatures may require this basic thermoregulatory technology even in otherwise warm climates. The elevated prevalence of mats in warmer environments may reflect comfort, privacy/soundproofing, and decoration these items have to offer in the general household rather than their provision of

warmth. Additionally, warmer environments are related to increased frequency and intensity of disease, pathogens, and insect pests (National Research Council 2001). Therefore, floormats provide the opportunity for individuals to avoid pests and stay off the ground to maintain hygiene and health. Finally, floormats can provide a cooling effect associated with areas where heat can become trapped underground (García-García et al. 2023).

The higher proportion of basketry production within warmer environments could be reflective of the availability of seeds, berries, and other plants collected for subsistence considering there is evidence of using basketry as a specialized food-processing technique (Geib and Jolie 2008; Supplemental Appendix A). The quality and quantity of plant materials for basketry manufacture is more abundance in temperature climates and may also explain the observed pattern. Future research may evaluate different baskets making plant materials described in the eHRAF World Cultures database and determine distributions are correlated with warm or cold temperatures.

The categories of ceremonies/rituals, storytelling, and medical care observed as part of this research are not correlated with temperature. This is not surprising considering the pancultural nature of such activities (LeGare and Watson-Jones 2015; Shynkaruk et al. 2018; Wong et al. 2014). Tattooing and piercing are additional perforator tool activities with no correlation with temperature. While initially we expected that tattooing would be associated with warmer climates where skin tends to be more visible, there are many accounts of tattooing areas of body that are more visible to others even while clothing is worn such as the face, hands, and neck (see Supplemental Appendix A). It is also clear that tattoos have personal significance which helps to explain why needles are used to tattoo portions of the body that would be covered by clothing while exposed to cold external environments such as the arms, chest, shoulder, back,

and legs (see Supplemental Appendix A). In other words, exposing large portions of the skin to external environmental conditions is not necessary for tattoos to carry significance, thus tattooing can occur in both warm and cold climates.

Conclusions and Implications for Archaeological Record

This study examined a hypothesized relationship between environmental temperature and perforator tool use in human societies by examining the prevalence of needle and awl technologies among 59 distinct North American Indigenous groups occupying variable climatic conditions. The data support the hypothesized increased dependence on perforator tools in cold temperatures. This research is one of the first to quantitatively estimate the effect of temperature on perforator tool use in the ethnographic record. The manufacture of clothing is an important motivator for the creation and use of needles and awls. While the data are consistent with this model, this study furthermore underscores the multifaceted utility of perforator tools across diverse cultural contexts.

As a proxy for interpreting perforator tools in the past, the findings presented here anticipate that the prevalence of Paleolithic bone needles and awls in archaeological contexts should increase in colder climates. The data also imply that while clothing manufacture and maintenance is likely a major driver of perforator tool presence in the archaeological record, the presence of perforator tools does not guarantee their use as thermoregulatory technologies. Researchers should proceed with caution when evaluating needles and awls in archaeological contexts and avoid preconceived notions that these tools act as direct proxies for the manufacture of thermoregulatory material culture in the past. A more appropriate and tempered approach should acknowledge the multiplicity of behaviors associated with such tools. Nonetheless, a quantitative approach in which analysts consider not only the presence of perforator tools but

also their abundance in the archaeological record should be used to strengthen inferences about thermoregulatory activities in a given region in the past. A future archaeological analysis of the relationship between temperature and perforator occurrence across North America would certainly offer a complimentary approach toward further clarifying the relationship between climatic conditions and perforator tool use in human societies.

Acknowledgments: We thank Allison Caine for providing insight into the ethnographic explanations for observed temperature relationships and perforator tool use. The authors also thank Bree Doering and Todd Surovell for reading earlier drafts of the paper. The ethnographic data analyzed for this project was provided by the eHRAF World Cultures database. Finally, this manuscript greatly benefits from comments provided by the editors and anonymous reviewers.

Author Contributions Statement: McKenna L. Litynski contributed to theoretical development, ethnographic meta-analysis data compilation, manuscript preparation and revisions, and statistical analyses. Sean Field contributed to statistical analyses and manuscript revisions. Randall Haas contributed to theoretical development, statistical analyses, and manuscript revisions.

Funding Statement: No grants, scholarships, or alternative sources of funding supported this project.

Data Availability Statement: The data generated for this analysis are available in the text and supplemental materials.

Competing Interests: The authors declare none.

Supplemental Material

Supplemental Appendix A: Excel spreadsheet featuring the raw data compiled for this study based texts available within the eHRAF World Cultures database.

References Cited:

Allison PA, Bottjer DJ. Taphonomy: Bias and Process Through Time. In: Allison PA, Bottjer DJ, editors. Taphonomy. Aims and Scope Topics in Geobiology Book Series, vol 32. Dordrecht: Springer; 2010.

Bates J, Needham A, Conneller C, Milner N, Pomstra D, Little A. Flint awls at the Mesolithic site of Star Carr: Understanding tool use through integrated methods. J Archaeol Sci Rep. 2022;43:103478.

Breitborde R. A Functional Bone Tool Analysis [M.A. Thesis]. Northridge (CA): California State University, Department of Anthropology; 1983.

Binford LR. Willow Smoke and Dogs' Tails: Hunter-Gatherer Settlement Systems and Archaeological Site Formation. Am Antiquity. 1980;45(1):4-20.

Blomberg NJ. Sacred Figures in Navajo Blankets: A Re-Evaluation of Timeframe and It's Implication for Cultural Change. J Southwest Anthropol Hist. 2016;55(4):357-72.

De Laguna F. Under Mount Saint Elias: The History and Culture of the Yakutat Tlingit. In: Smithsonian Contributions to Anthropology, vol. 7. Washington, D.C.: Smithsonian Institution Press; 1972. Available from: https://ehrafworldcultures-yale-edu.libproxy.uwyo.edu/document?id=na12-020

Dean LG, Vale GL, Laland KN, Flynn E, Kendal RL. Human cumulative culture: a comparative perspective. Biol Rev. 2014;89(2):284-301.

Denys C. Taphonomy and Experimentation. Archaeometry. 2002;44(3):463-84.

Divale WT. Female status and cultural evolution: A study in ethnographer bias. Behav Sci Res. 1976;11(3):169-211.

d'Errico F, Doyon L, Zhang S, Baumann M, Lázničková-Galetová M, Gao X, et al. The origin and evolution of sewing technologies in Eurasia and North America. J Hum Evol. 2018;125:75-86.

d'Errico F, Backwell LR, Wadley L. Identifying regional variability in Middle Stone Age bone technology: The case of Sibudu Cave. J Archaeol Sci. 2012;39:2479-95.

Densmore F. Chippewa Customs. Bulletin, no. 86. U.S. Govt. print. off.; 1929. p. xii, 209, plates. Available from: https://ehrafworldcultures.yale.edu/document?id=ng06-005

Fick SE, Hijmans RJ. WorldClim 2: new 1km spatial resolution climate surfaces for global land areas. International Journal of Climatology. 2017;37(12):4302-15.

Fischer MD, Ember CR. Big Data and Research Opportunities Using HRAF Databases. In: Chen S-H, editor. Big Data in Computational Social Science and Humanities. New York, NY: Springer; 2018.

García-García A, Cuesta-Valero FJ, Miralles DG, Mahecha MD, Quaas J, Reichstein M, et al. Soil heat extremes can outpace air temperature extremes. Nat Clim Change. 2023;13:1237-41.

Garvey R. Patagonian Prehistory: Human Ecology and Cultural Evolution in the Land of Giants. Salt Lake City (UT): University of Utah Press; 2021.

Geib PR, Jolie EA. The Role of Basketry in Early Holocene Small Seed Exploitation: Implications of a ca. 9,000 Year-Old Basket from Cowboy Cave, Utah. Am Antiquity. 2008;73(1):83-102.

Gijn AL van. Implements of bone and antler: A Mesolithic tradition continued. In: Kooijmans LPFB Louwe, editor. Schipluiden: a neolithic settlement on the dutch north sea coast c. 3500 cal bc. Leiden: Faculty of Archaeology, Leiden University; 2006. p. 207-24.

Gilligan I. The Prehistoric Development of Clothing: Archaeological Implications of a Thermal Model. J Archaeol Theory. 2010;17:15-80.

Gilligan I, d'Errico F, Doyon L, Wang W, Kuzmin YV. Paleolithic eyed bone needles and the evolution of dress. Sci Adv. 2024;10(26).

Hayes AC, Lancaster J. Badger House Community. Mesa Verde National Park, Archaeological Research Series 7E. 1975.

Henshilwood CS, d'Errico F, Marean CW, Milo RG, Yates R. An early bone tool industry from Middle Stone Age at Blombos Cave, South Africa: implications for the origins of modern human behaviour, symbolism, and language. J Hum Evol. 2001;41:631-78.

Jenness D. The Material Culture of the Copper Eskimos. Report of the Canadian Arctic Expedition, 1913-1918, Southern Party, 1913-1916, vol. 16. Edmond Cloutier, King's Printer and Controller of Stationery; 1946. p. 8, 148. Available from: https://ehrafworldcultures.yale.edu/document?id=nd08-028

Keddie G. Bone Awls: Bridging or Widening the Gaps between Archaeology and Ethnology. The Midden. 2016;44(1).

Kelly RL. Hunter-Gatherer Mobility Strategies. J Anthropol Res. 1983;39(3):277-306.

Kelly RL. The Lifeways of Hunter-Gatherers: The Foraging Spectrum. 2nd ed. Cambridge: Cambridge University Press; 2013.

Khau M. Gender and the politics of the Basotho blanket. In: Moletsane R, Mitchell C, Smith A, editors. Was it something I wore? Dress Identity Materiality. Cape Town, South Africa: HSRC Press; 2012.

LeGare CH, Watson-Jones RE. The Evolution and Ontogeny of Ritual. In: Buss DM, editor. The Handbook of Evolutionary Psychology, Volume 2: Integrations. 2nd ed. Hoboken (NJ): John Wiley and Sons; 2015.

Lycett SJ. Spatially mediated cultural transmission and the distribution of horse blanket styles among post-contact and reservation-period tribes of western North America. Archaeol Anthropol Sci. 2020;12(5).

Loffler G. Subsistence-patterns, gender roles, effective temperature, and the evolutionary timing of a post reproductive life span. Med Hypotheses. 2016;89:48-57.

Lyman RL. North American Paleoindian Eyed Bone Needles: Morphometrics, Sewing, and Site Structure. Am Antiquity. 2015;80(1):146-60.

Madgwick R, Mulville J. Reconstructing depositional histories through bone taphonomy: extending the potential of faunal data. J Archaeol Sci. 2015;53:255-63.

McIlwraith TF. The Bella Coola Indians: Volume Two. University of Toronto Press; 1948. Available from: https://ehrafworldcultures.yale.edu/document?id=ne06-002

National Research Council (US) Committee on Climate, Ecosystems, Infectious Disease, and Human Health. Under the Weather: Climate, Ecosystems, and Infectious Disease. Washington DC: National Academies Press (US); 2001.

Osbora AJ. Eye of the Needle: Cold Stress, Clothing, and Sewing Technology during the Younger Dryas Cold Event in North America. Am Antiquity. 2014;79(1):45-68.

Pearlstein E. Restoring provenance to a Native American feather blanket. Museum Manag Curatorship. 2008;25(1).

Pelton S. A Thermoregulatory Framework for Hominin Research: Archaeological and Ethnographic Studies with Implications for Global Human Dispersal [Ph.D. dissertation]. Laramie (WY): University of Wyoming, Department of Anthropology; 2019.

Pelton S, Litynski M, Allaun S, Buckley M, Govaerts J, Schoborg T, et al. Early Paleoindian use of canids, felids, and hares for bone needle production at the La Prele site, Wyoming, USA. PLoS ONE. 2024;19(11):e0313610.

Rapp Py-Daniel A. Bones: Preservation and Conservation. In: Smith S, editor. Encyclopedia of Global Archaeology. New York, NY: Springer; 2014.

Roberts F. Shabik'eshchee Village: A Late Basketmaker Site in the Chaco Canyon, New Mexico. Bureau of American Ethnology Bulletin No. 92. Smithsonian Institution; 1929.

Shaffer BS. Quarter-Inch Screening: Understanding Biases in Recovery of Vertebrate Faunal Remains. Am Antiquity. 1992;57(1):129-36.

Shynkaruk VD, Salata G, Danilova T. Myth as a Phenomenon of Culture. Natl Acad Managerial Staff Cult Arts Herald. 2018;UDC 291.13:130.2.

Slocum S. Woman the gatherer: male bias in anthropology. In: Reiter RR, editor. Toward an Anthropology of Women. New York and London: Monthly Review Press; 1975.

Stewart KM, Wigen RJ. Screen Size and the Needle for Reinterpretation: A Case Study from the Northwest Coast. Bull Fla Mus Nat His. 2003;44(1):27-34.

Torgerson R. Methodological Biases in Zooarchaeology and the Interpretation of Human Activity from the Faunal Record. Student Anthropol. 2013;3(3):81-92.

Watts J, Jackson JC, Arnison C, Hamerslag EM, Shaver JH, Purzycki BG. Building Quantitative Cross-Cultural Databases From Ethnographic Records: Promise, Problems, and Principles. SAGE Publ Cross-Cult Res. 2021;56(1):62-94.

Whitaker K. Navajo Saddle Blankets: Textiles to Ride in the American West. Am Indian Cult Res J. 2004;28(2).

Wobst MH. The Archaeo-Ethnology of Hunter-Gatherers or the Tyranny of the Ethnographic Record in Archaeology. Am Antiquity. 2017;43(2):303-9.

Wang L, Li T, Wang Q, Tong Y, Chen S, Yuan S, et al. Ancient DNA insights into Neolithic bone-tool use on the Tibetan Plateau. J Archaeol Sci. 2025;177:106183.

Wong YS, Allotey P, Reidpath DD. Health Care as Commons: An Indigenous Approach to Universal Health Coverage. Int Indigenous Policy J. 2014;5(3).

Figure 1

Click here to access/download;Figure;Figure1.tif

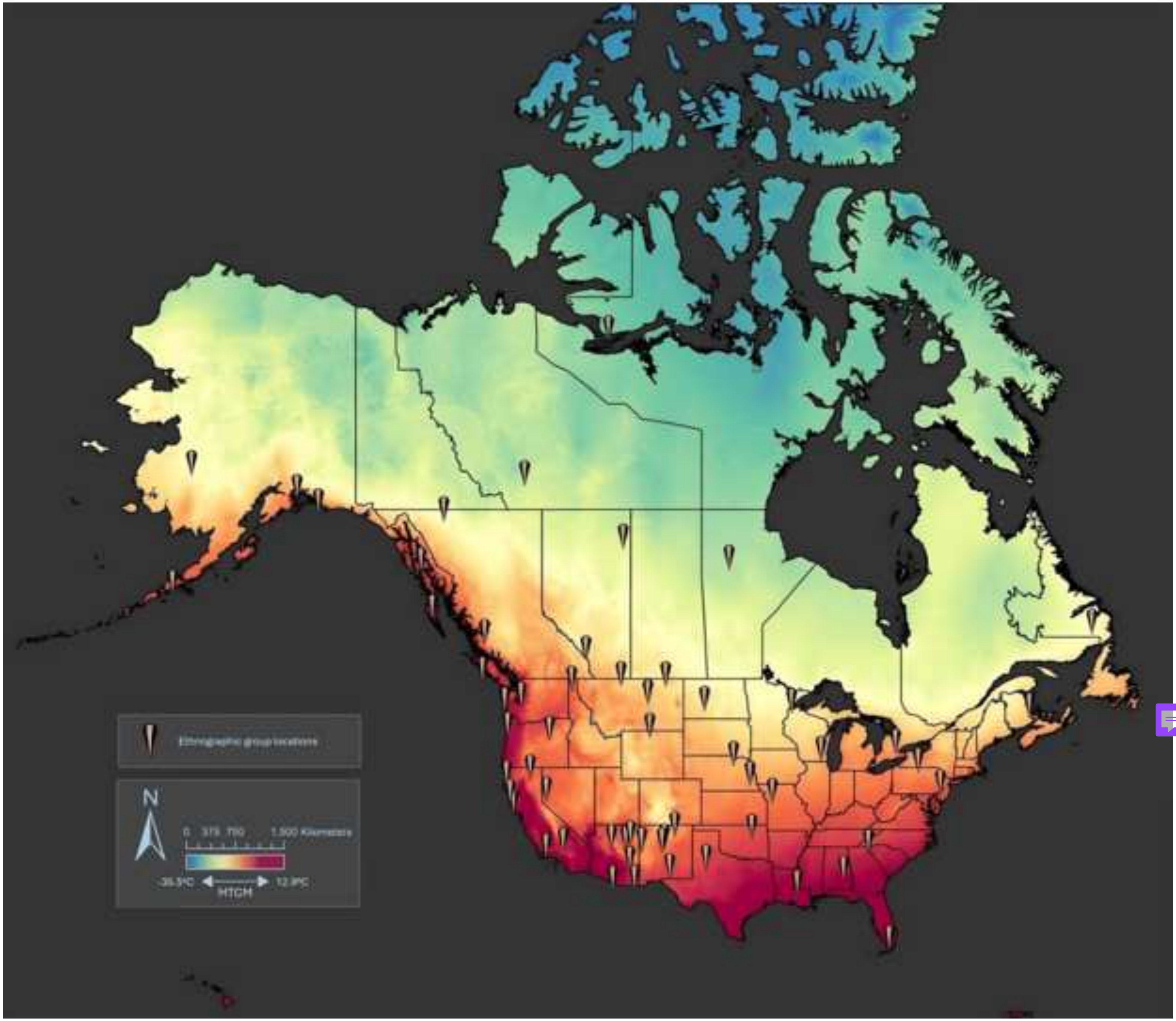

Figure 2

Click here to access/download;Figure;Figure2.tif

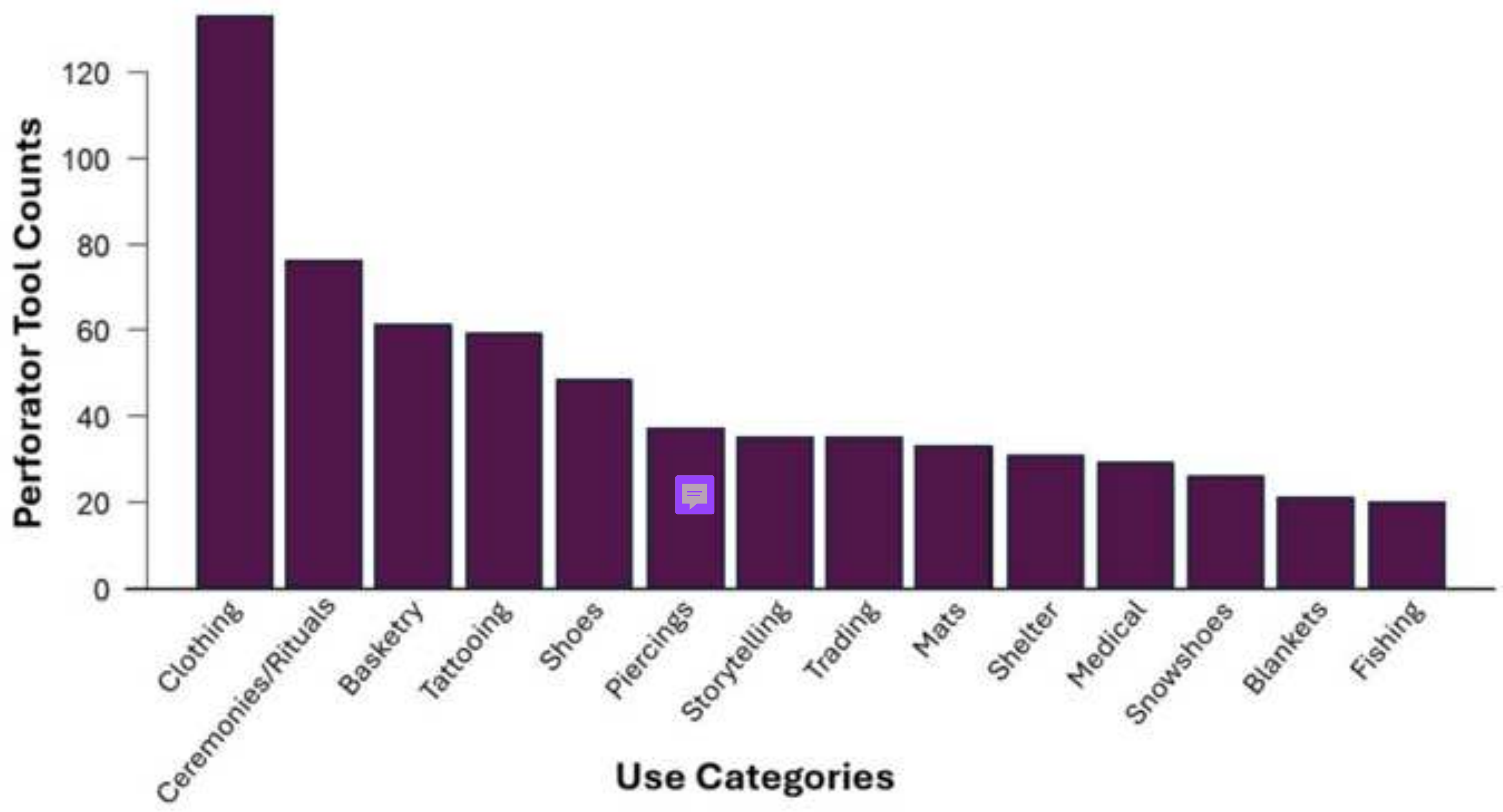

Figure 3

Click here to access/download;Figure;Figure3.tif

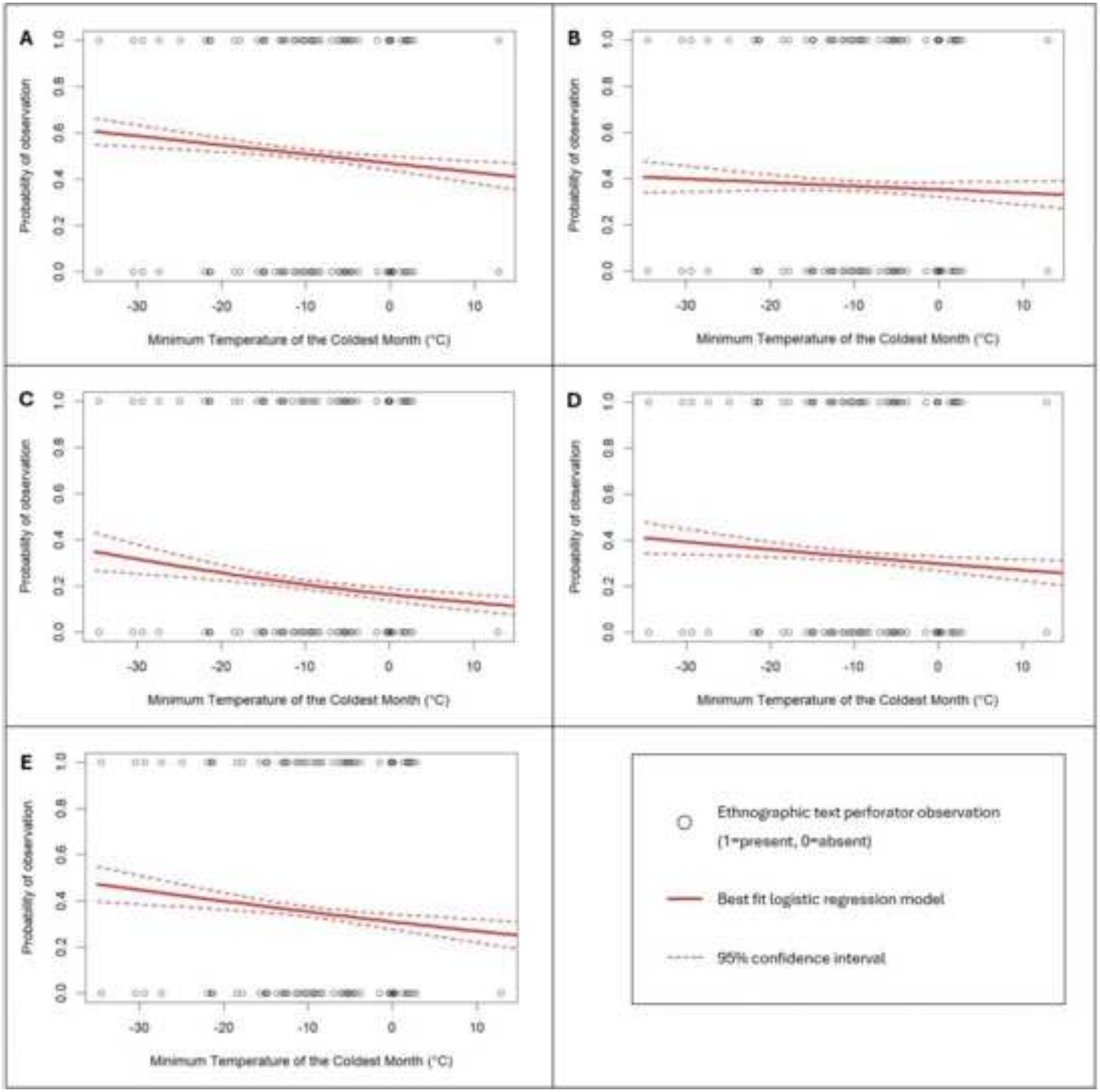

Click here to access/download
Supporting Information
Supplemental Materials_Appendix A.xlsx
